# Phospholipase D Toxins of Brown Spider Venom Convert Lysophosphatidylcholine and Sphingomyelin to Cyclic Phosphates

**DOI:** 10.1371/journal.pone.0072372

**Published:** 2013-08-29

**Authors:** Daniel M. Lajoie, Pamela A. Zobel-Thropp, Vlad K. Kumirov, Vahe Bandarian, Greta J. Binford, Matthew H. J. Cordes

**Affiliations:** 1 Department of Chemistry and Biochemistry, University of Arizona, Tucson, Arizona, United States of America; 2 Department of Biology, Lewis and Clark College, Portland, Oregon, United States of America; Consejo Superior de Investigaciones Cientificas, Spain

## Abstract

Venoms of brown spiders in the genus *Loxosceles* contain phospholipase D enzyme toxins that can cause severe dermonecrosis and even death in humans. These toxins cleave the substrates sphingomyelin and lysophosphatidylcholine in mammalian tissues, releasing the choline head group. The other products of substrate cleavage have previously been reported to be monoester phospholipids, which would result from substrate hydrolysis. Using ^31^P NMR and mass spectrometry we demonstrate that recombinant toxins, as well as whole venoms from diverse *Loxosceles* species, exclusively catalyze transphosphatidylation rather than hydrolysis, forming cyclic phosphate products from both major substrates. Cyclic phosphates have vastly different biological properties from their monoester counterparts, and they may be relevant to the pathology of brown spider envenomation.

## Introduction

Envenomation by brown spiders in the genus *Loxosceles* can induce a disease state called loxoscelism in mammalian tissue. Cutaneous loxoscelism can result in ulcer formation, edema, and dermonecrosis at the site of envenomation. Some envenomations result in systemic loxoscelism involving hemolysis, circulatory shock, intravascular coagulation, renal failure and even death [1]–[4].

Phospholipase D (PLD) toxins in the venom are the primary agents responsible for loxoscelism**.** Toxins purified from venom or recombinant sources elicit the full pathology of loxoscelism when injected into animal models [5]–[7]. Multiple PLD isoforms and homologs are expressed in venoms throughout the spider family Sicariidae, which includes the genera *Loxosceles* and *Sicarius.* The gene family comprising these toxins has been named *SicTox* to reflect this phylogenetic distribution [8].

PLD toxins from *Loxosceles* bind to mammalian cell surfaces [9] and have enzymatic activity against common phospholipids in mammalian tissue, including lysophosphatidylcholine (LPC) and sphingomyelin (SM) [2], [4], [10]. The most common activity assay for these enzymes detects PLD activity by monitoring choline release from substrate. Liberation of choline from SM or LPC is commonly assumed to result from substrate hydrolysis, giving either ceramide-1-phosphate (C1P) or lysophosphatidic acid (LPA), respectively, as a second product [10]–[14]. C1P and LPA are thought to contribute to the pathology of loxoscelism [14], [15] by some as yet undetermined mechanism [16], but definitive evidence for formation of these phosphate-containing products is lacking. Here we use ^31^P NMR spectroscopy and mass spectrometry to directly observe formation of the phosphate-containing products from the action of a recombinant *Loxosceles* PLD toxin, as well as diverse whole venom samples, on the substrates SM and LPC. Instead of the hydrolytic products C1P and LPA, respectively, we observe exclusive formation of cyclic phosphate products resulting from intramolecular transphosphatidylation.

## Materials and Methods

### Materials

Natural (purified from chicken egg) and synthetic versions of LPC and SM were purchased from Avanti Polar Lpids (Alabaster, Alabama, USA). Synthetic lipids are: hexanoyl SM (6∶0 SM, N-hexanoyl-D-*erythro*-sphingosylphosphorylcholine), octanoyl LPC (1-octanoyl-2-hydroxy-*sn*-glycero-3-phosphocholine), palmitoyl LPC (1-palmitoyl-2-hydroxy-*sn*-glycero-3-phosphocholine), palmitoyl LPA (1-palmitoyl-2-hydroxy-*sn*-glycero-3-phosphate), and palmitoyl CPA (1-palmitoyl-*sn*-glycero-2,3-cyclic-phosphate). QuikChange Site-Directed Mutagenesis Kit was purchased from Stratagene (La Jolla, California, USA). BugBuster and Benzonase Nuclease were purchased from Novagen (Madison, Wisconsin). Ni-NTA Spin Columns were purchased from Qiagen (Hilden, Germany). Amplex Red Sphingomyelinase Assay Kit was purchased from Invitrogen (Carlsbad, California, USA). Flat bottom 96-well plates were obtained from Costar. NMR tubes were purchased from Wilmad-LabGlass (Vineland, New Jersey, USA). All other chemicals were purchased from standard sources.

### Recombinant Expression and Purification of αIB2bi

DNA encoding mature LaSicTox-αIB2bi (gb:AY699703) was previously cloned into a pHIS8 bacterial expression vector [17]. An inactive H47N variant of this construct was generated by QuikChange site-directed mutagenesis. N-terminally histidine-tagged recombinant proteins were expressed from these constructs in *Escherichia coli* strain BL21(λDE3) closely following published methods [18]. Growth medium (50 mL 2×YT containing 30 µg/mL kanamycin) was inoculated with freshly transformed cells and incubated at 37°C with shaking at 250 rpm. When the culture reached OD_600_∼0.6, expression was induced by addition of IPTG to a working concentration of 0.1 mg/mL. After 2 h, the induced cells were pelleted by centrifugation at 5,000*×g* for 10 min at 4°C. Cell pellets were resuspended in BugBuster lysis reagent (5 mL/g wet cell paste) containing Benzonase Nuclease (1 µL per mL of lysate) [17], incubated at room temperature for 20 min to allow lysis, and then centrifuged at 16,000*×g* to remove insoluble material. The cleared lysate was brought to a concentration of 20 mM imidazole by addition of NE250 buffer (0.1 M Tris [pH 8], 0.2 M NaCl and 250 mM imidazole), and then loaded in two 0.6 mL portions onto a Qiagen Ni-NTA spin column that had been equilibrated in NW20 buffer (0.1 M Tris [pH 8], 0.2 M NaCl, and 20 mM imidazole). Flow-through fractions (FT) were collected by centrifuging the spin columns at 250×*g* for 5 min. The column resin was washed twice with 0.6 mL of NW20 buffer. Wash fractions were collected by centrifugation at 850×*g* for 2 min. Protein was eluted from the resin with two 0.3 mL aliquots of NE250 buffer at 250×*g* for 5 min. Eluates that contained >90% pure, active enzyme as judged by Coomassie-stained SDS-PAGE and colorimetric PLD assays (see below and Figure S1), were brought to 50% (v/v) glycerol, divided into 20 µL aliquots, and stored at −20°C until needed.

### Colorimetric PLD Assay of Wild-type αIB2bi and H47N Variant

An Amplex Red Sphingomyelinase Assay Kit was used to screen preparations of recombinant protein for PLD activity. Fractions from affinity purification (100 µL) were pipetted into 96 well plates. An assay solution was prepared according to the kit instructions, except that alkaline phosphatase, which is not required for measurement of PLD activity with this kit, was omitted. Each sample of assay solution contained either sphingomyelin substrate at a working concentration of 0.25 mM in mixed micelles with Triton X-100 detergent, or an equivalent concentration of palmitoyl lysophosphatidylcholine substrate without Triton X-100. Assay solution (100 µL) was added to each protein sample or fraction and the plate was incubated in the dark at 37°C for 1 h. Choline release/PLD activity was monitored by the appearance of the pink color of resorufin.

### Venom Extraction/Species Determination

Venom was extracted from mature females of *L. arizonica, L. reclusa* and *L. laeta,* with species confirmation by morphology and COI barcodes. The animals were collected live from the respective localities of Yarnell, Arizona; Bumpass, Virginia; and Los Angeles, California (*L. laeta* are native to South America). In each case, owners gave permission for studies to be conducted on site. Vouchers are stored in the lab of GJB and detailed collecting information is available on request. Venom was extracted using electrostimulation taking care to avoid contamination from digestive enzymes [8]. Venom samples were stored frozen at −70°C.

### ^31^P-NMR

^31^P NMR spectra were recorded at 298 K on a Bruker DRX-500 spectrometer equipped with a BBO-500 MHz S2 5 mm probe with Z gradient. Spectra were acquired at a spectrometer frequency of 202.11 MHz with ^1^H decoupling. Data points (32 K) were acquired per spectrum at a spectral width of 80 ppm, with signal averaging over 200 scans. A 60° pulse length and a 7 s relaxation delay were employed to enable quantitative resonance integration [19]. Substrate micelle samples included either 0.1 M octanoyl LPC and 10 mM trimethyl phosphate (TMP) in borate buffer (100 mM borate [pH 8], 10 mM MgCl_2_, 10% D_2_O); 4 mM palmitoyl LPC and 1 mM TMP in borate buffer; or 4 mM hexanoyl SM, 4 mM Triton X-100, and 1 mM TMP in borate buffer. Each substrate micelle sample (0.5 mL volume) was added to a 5 mm thin wall NMR sample tube and an initial spectrum was recorded in the absence of enzyme or venom. For recombinant enzyme assays, 20 µL of enzyme glycerol stock was added to the lipid sample. For venom assays, extracted venom was reconstituted in 20 µL 1× RB buffer (100 mM Tris [pH 7.4], 10 mM MgCl_2_) and 5 µL of reconstituted venom solution was added to the lipid sample. Between spectral acquisitions, all reactions were stored either in the magnet at 298 K or at ambient temperature. Data were processed and analyzed with MestReNova 7.1.1 (Mestrelab Research, Santiago de Compostela, Spain). A baseline correction was applied to the data and the chemical shifts were referenced to TMP at 3.02 ppm [20]. Quantitative peak integration was performed with the autodetect function in MestReNova and the integrated peak areas were normalized to the concentration of the TMP standard. Percentage yields of product over time (as well as percentages of substrate remaining) were calculated by dividing the normalized integrated peak area of product (or substrate) resonance by the normalized integrated peak area of the substrate resonance before enzyme addition.

### LC MS/MS Analysis of Reaction Products

**Figure 1:**
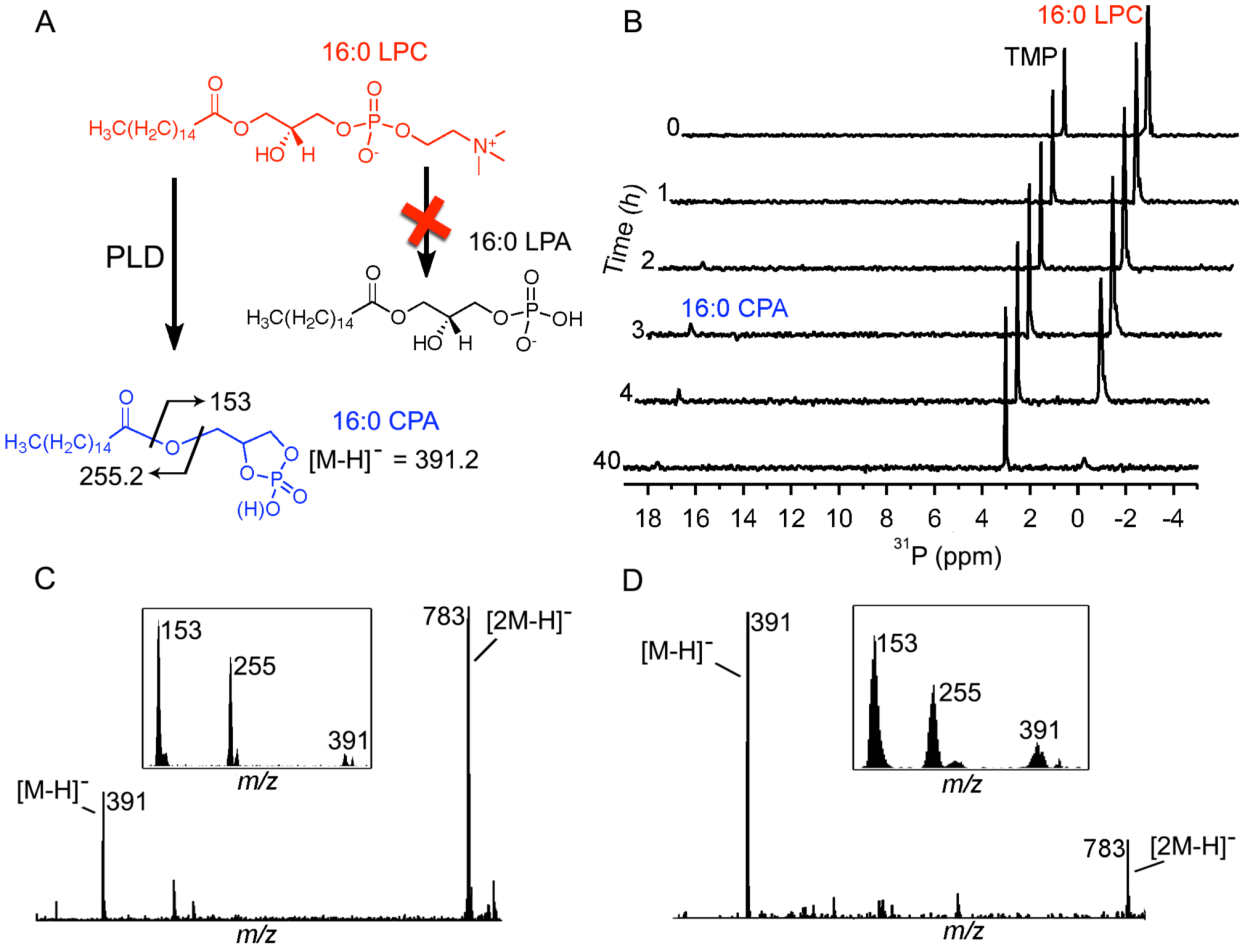
Action of recombinant SicTox PLD enzyme αIB2bi on palmitoyl lysophosphatidylcholine (16∶0 LPC). (A) No palmitoyl lysophosphatidic acid (16∶0 LPA) is detected, but palmitoyl cyclic phosphatidic acid (16∶0 CPA) can be detected as a product by NMR and confirmed by MS/MS based on known ion fragmentation patterns of 16∶0 CPA [21], [22]. (B) Degradation of 16∶0 LPC by SicTox enzyme αIB2bi as measured by ^31^P-NMR. The only observed product resonance (+17.2 ppm) is characteristic of a cyclic phosphate species with a five-membered ring, matches the chemical shift of commercially available 16∶0 CPA, and is inconsistent with the chemical shift of 16∶0 LPA (see Figure S2). After 40 h, nearly all the LPC substrate is consumed, but the putative CPA resonance remains weak, presumably due to poor solubility. Trimethyl phosphate (TMP; 1 mM) was added as a chemical shift and concentration standard (see Materials and Methods). (C) Mass spectrum of 16∶0 CPA standard showing [M-H]^-^ monomer at *m/z* = 391 as well as the [2M-H]^−^ dimer at *m/z* = 783. Inset shows the MS/MS fragmentation of the *m/z* 391 species, yielding the daughter ions depicted in (A). (D) Mass spectrum of extracted reaction mixture of palmitoyl LPC substrate treated with αIB2bi enzyme showing the same [M-H]- and [2M-H]- species as in (C). Inset shows the MS/MS fragmentation of *m/z* 391 which yields the same daughter ions as the 16∶0 CPA standard in (C).

Lipids were isolated from NMR samples for LC-MS/MS as follows [21]: the NMR sample (0.5 mL) was combined with 2 mL of a 2∶1 (v/v) methanol:chloroform mixture, vortexed, and incubated for 10 min at 25°C. The resulting mixture was centrifuged at 3,000×*g* for 10 min at 10°C. The top layer was removed from the solution and then evaporated under nitrogen gas. The resulting residue was brought up in methanol containing 0.1 M (NH_4_)_2_SO_4_. Aliquots (2–10 µL) were injected onto an Agilent Eclipse XDB-C18 column (5 µm, 4.6×250-mm) equilibrated in water and operating at flow rate of 0.2 mL/min. A linear gradient from 0 to 100% acetonitrile was applied over 20 min, followed by 15 min at 100% acetonitrile prior to re-equilibrating the column in water. The eluate was analyzed by an in-line electrospray (ES) ionization-equipped LCQ ThermoFinnigan Deca XP mass spectrometer, which was operated both in negative and positive ion mode as necessary for each run. The *m/z* range of 300–2000 atomic mass units was scanned and the ES was set to 4.5 kV ionization energy in positive ionization mode and 3 kV in negative ionization energy. The source temperature was 350°C. Fragmentation experiments were carried out by isolating the desired mass and applying a collision energy of 35%. For the 16∶0 chain lyso-lipids, MS was performed by direct infusion. For these experiments, palmitoyl LPC was digested by αIB2bi as described in Figure 1. Lipids were extracted as described above. The lipid residue was dissolved to a concentration of 50–80 µM in a 1∶1 H_2_O:acetonitrile mixture containing 0.1% TFA. An aliquot of commercially available 16∶0 CPA in chloroform was dried under nitrogen, resuspended in the same solution and treated as a standard. These solutions were then infused into the electrospray ionization (ESI) source of a Thermoelectron LCQ Classic ion trap instrument. The ESI source was operated in the negative ion mode under standard tuning conditions with a spray voltage 4.5 kV and a capillary temperature of 200°C. Tandem MS/MS experiments were also carried out for the selected precursor [M-H]^-^ at *m/z* 391. The collision energy was kept at an arbitrary value of 28% in each experiment.

## Results

### Lysis of Palmitoyl LPC by PLD Toxin αIB2bi from *Loxosceles arizonica* Yields Cyclic Phosphatidic Acid as the Only Observed Phosphate-containing Product

We expressed and purified a recombinant SicTox enzyme from *L. arizonica* (αIB2bi) [17] (Figure S1A) and verified activity against palmitoyl (16∶0) LPC using an enzyme-linked colorimetric assay (Figure S1C) and ^31^P NMR (Figure 1). The colorimetric assay showed production of choline and the ^31^P NMR assay showed clear loss of the LPC resonance at −0.5 ppm. As a negative control, an inactive H47N variant of αIB2bi did not release choline from LPC (Figure S1C), and did not diminish the substrate ^31^P NMR resonance.

Despite clear consumption of substrate and formation of choline, we saw no evidence for formation of LPA in the ^31^P NMR assay. Instead, we observed appearance of a weak unidentified resonance at +17.2 ppm, the intensity of which accounted for <10% of the lost substrate signal (Figure 1B). A white precipitate also formed during the reaction. Mixed micelles of commercially available palmitoyl LPC and LPA exhibited resonances at +3.4 ppm for LPA and did not form precipitates (Figure S2C). These results suggest that the phosphate-containing product is not LPA.

We hypothesized that the enzyme might be catalyzing formation of a poorly soluble cyclic phosphate product. Indeed, the chemical shift of the NMR-visible product precisely matches that of a palmitoyl cyclic phosphatidic acid standard (16∶0 CPA) doped into LPC micelles (Figure S2D), and such mixed micelle samples do show precipitation. Moreover, ESI-MS and tandem MS/MS spectra of the enzyme reaction mixtures (Figure 1D) show close matches to ions and ion fragmentation patterns seen in mass spectra of 16∶0 CPA standards (Figure 1C) [21], [22]. These results confirm that 16∶0 CPA is a product of the reaction catalyzed by αIB2bi with LPC as substrate.

### *L. arizonica* αIB2bi Toxin Exclusively Produces Soluble CPA in High Yield from Short Acyl Chain LPC Substrates

**Figure 2:**
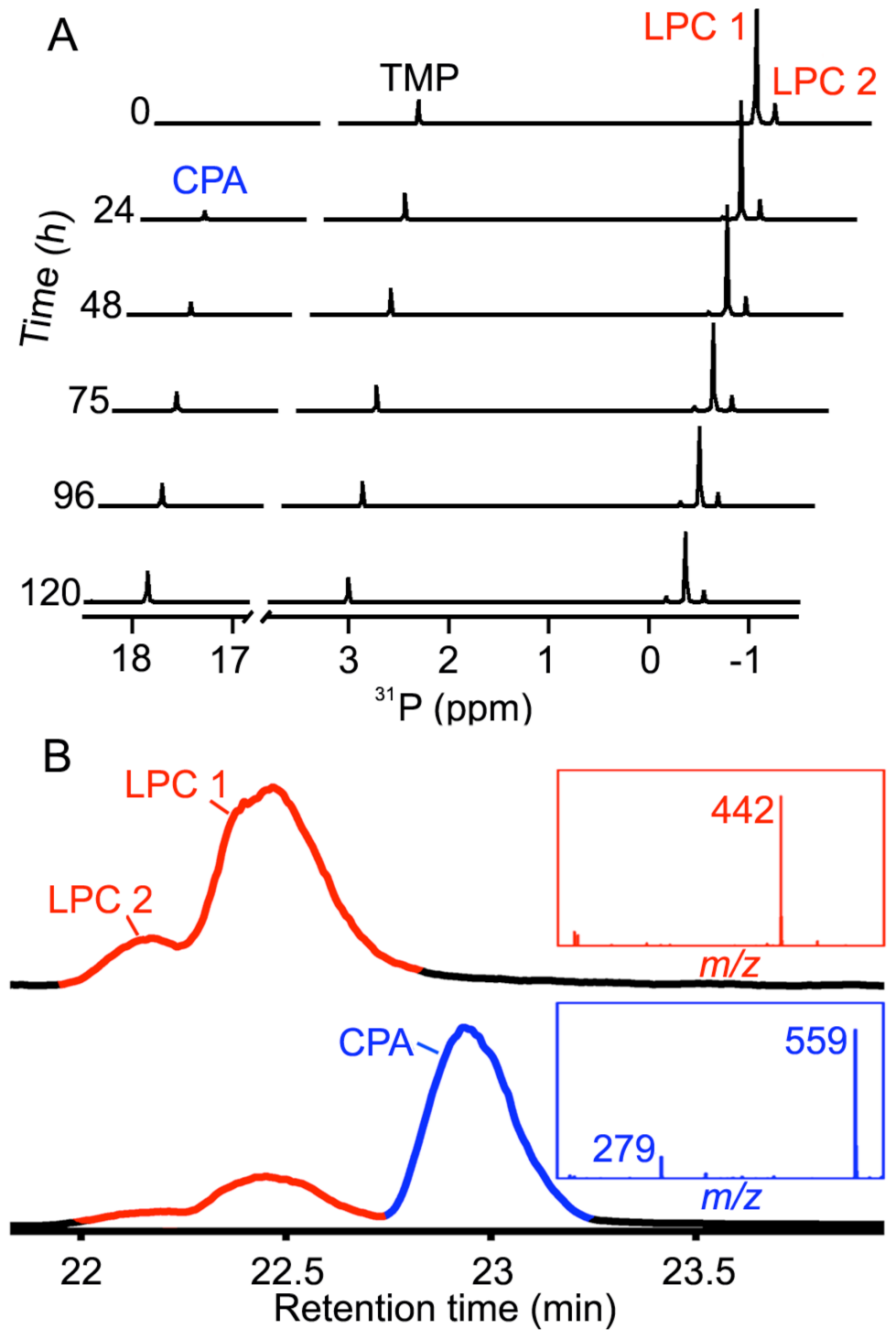
Recombinant SicTox enzyme αIB2bi generates cyclic phosphatidic acid from octanoyl lysophosphatidylcholine (8∶0 LPC). (A) Degradation of 8∶0 LPC by SicTox enzyme αIB2bi as measured by ^31^P-NMR. Two isomers of LPC (1 and 2) are observed (see text) and the only observed product upon enzyme addition is a far downfield chemical shift. Trimethyl phosphate (TMP) is an internal chemical shift and concentration standard. (B) LC-MS characterization of the NMR sample from (A), before (top) and after (bottom) addition of enzyme. Chromatograms represent total ion count (TIC) from reverse phase LC-MS as a function of retention time. Insets show time-averaged negative-ion mode mass spectra of substrate (red) and product peak (blue).

**Figure 3:**
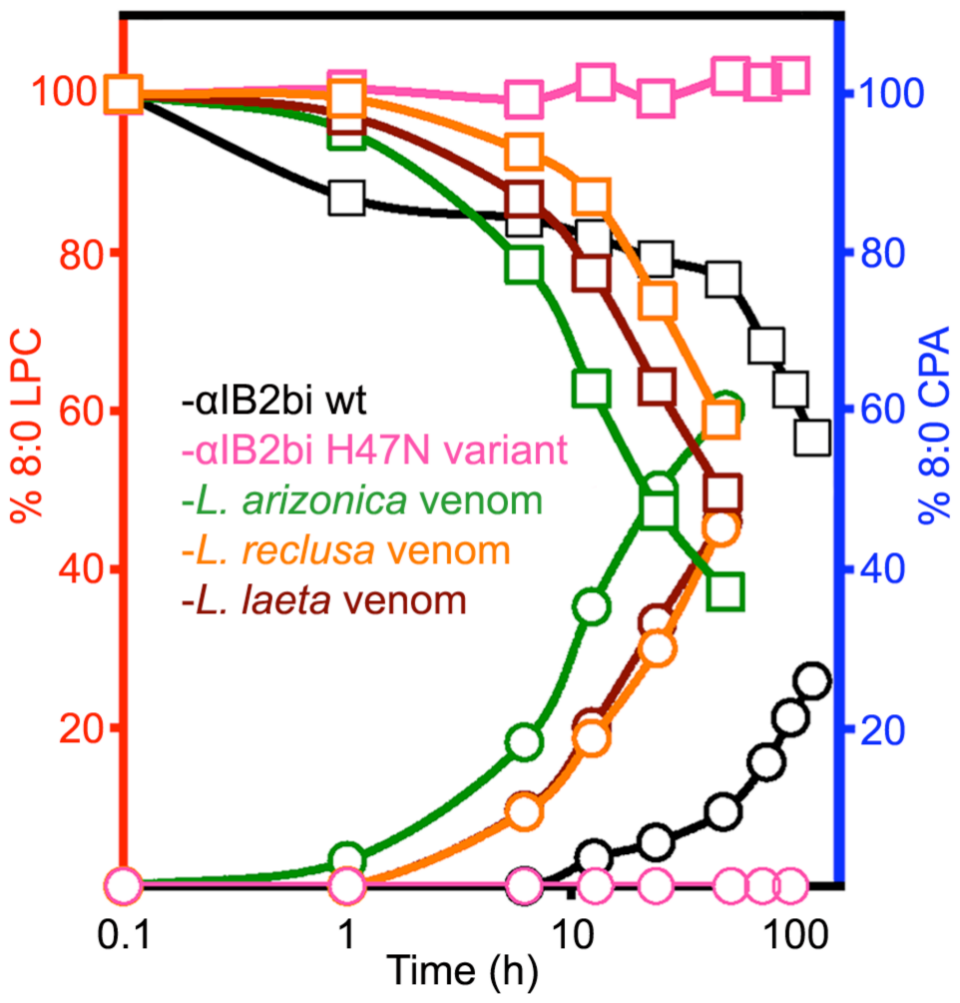
Diverse *Loxosceles* whole venoms cleanly convert 8∶0 LPC substrate to 8∶0 CPA product. Relative concentrations of 8∶0 LPC substrate (squares) and 8∶0 CPA product (circles) as a function of time at 298 K were measured by integration of ^31^P-NMR signals relative to a trimethyl phosphate standard (see Materials and Methods), and reported as percentages of the LPC concentration at time zero. Data points for the recombinant enzyme αIB2bi were derived from the experiment shown in Figure 2A, with the H47N variant shown as a negative control. Data for the three venoms were derived from similar experiments, but with addition of whole venom rather than purified recombinant enzyme.

*Loxosceles* PLD toxins can utilize lysophospholipids of various acyl chain lengths as substrates [10]. Thus, to improve product solubility, we repeated the ^31^P NMR assays with octanoyl (08∶0) LPC, a more soluble substrate (Figure 2). Under the assay conditions, octanoyl LPC exists as an equilibrium mixture of two isomers [23], 1-octanoyl-*sn*-glycero-3-phosphorylcholine (LPC 1) and 2-octanoyl-*sn*-glycero-phosphorylcholine (LPC 2), with LPC 1 predominating by a factor of ∼6. Incubation of αIB2bi with octanoyl LPC led to the appearance of species with a far downfield resonance similar to that observed when enzyme was added to palmitoyl LPC substrate (Figure 2A). With octanoyl LPC, however, loss of the substrate resonance is accompanied by gain of a comparable amount of product signal (see also Figure 3 for a quantitative analysis), and no precipitation is observed. The chemical shift of the product (+17.9 ppm) is clearly inconsistent with LPA, but agrees closely with the value reported for 1-octanoyl-glycero-2,3-cyclic-phosphate (08∶0 CPA) under similar reaction conditions [19].

We confirmed formation of 08∶0 CPA by analyzing reaction mixtures with LC MS/MS (Figure 2B). Negative ion mode-detected analysis of an aliquot of the substrate by reverse phase HPLC shows two peaks at 22.2 and 22.5 min, which are assigned to LPC 2 and LPC 1, respectively. The ratio of the two peak areas is consistent with the distributions observed by NMR. Both peaks contain a species with *m/z* = 442 corresponding to a [LPC+acetate]^–^ ion. Samples analyzed after addition of enzyme exhibit a new peak with a retention time of ∼23 min, the mass spectrum of which is dominated by two species with *m/z* of 279 and 559. The *m/z* = 279 peak can be assigned to [CPA]^–^. We assign the *m/z* = 559 species as the non-covalent [2CPA+H^+^]^–^ dimer, as it collapses cleanly to a single *m/z* = 279 species in MS/MS fragmentation. An analogous dimer also appeared in the 16∶0 CPA standard shown in Figure 1C. The NMR and MS data show unambiguously that under these conditions, recombinant *L. arizonica* αIB2bi enzyme catalyzes intramolecular cyclization of LPC to form CPA and choline exclusively. There is no detectable hydrolysis of substrate to form LPA.

### Whole *Loxosceles* Venoms also Convert LPC to CPA

We assayed whole venoms of three *Loxosceles* species (*L. laeta, L. reclusa, and L. arizonica*) from three geographically distinct regions to determine whether a similar activity is present in venom. Strikingly, all three whole venoms recapitulate the activity of the recombinant enzyme and form CPA in high yield as the sole ^31^P NMR-detectable product from LPC (Figure 3). Thus, the new activity is not an artifact of recombinant expression, presence of an affinity tag, or other such factors; nor does it result from some unusual feature of αIB2bi from *L. arizonica*. Exclusive production of CPA from LPC by venom is even more remarkable given that phylogenetically diverse venoms are known to contain multiple SicTox enzyme isoforms [8]. We conclude that formation of cyclic products must be a general feature of the SicTox enzymes from a variety of *Loxosceles* species.

### The SicTox PLD Toxins also Convert Sphingomyelin to a Cyclic Product

**Figure 4:**
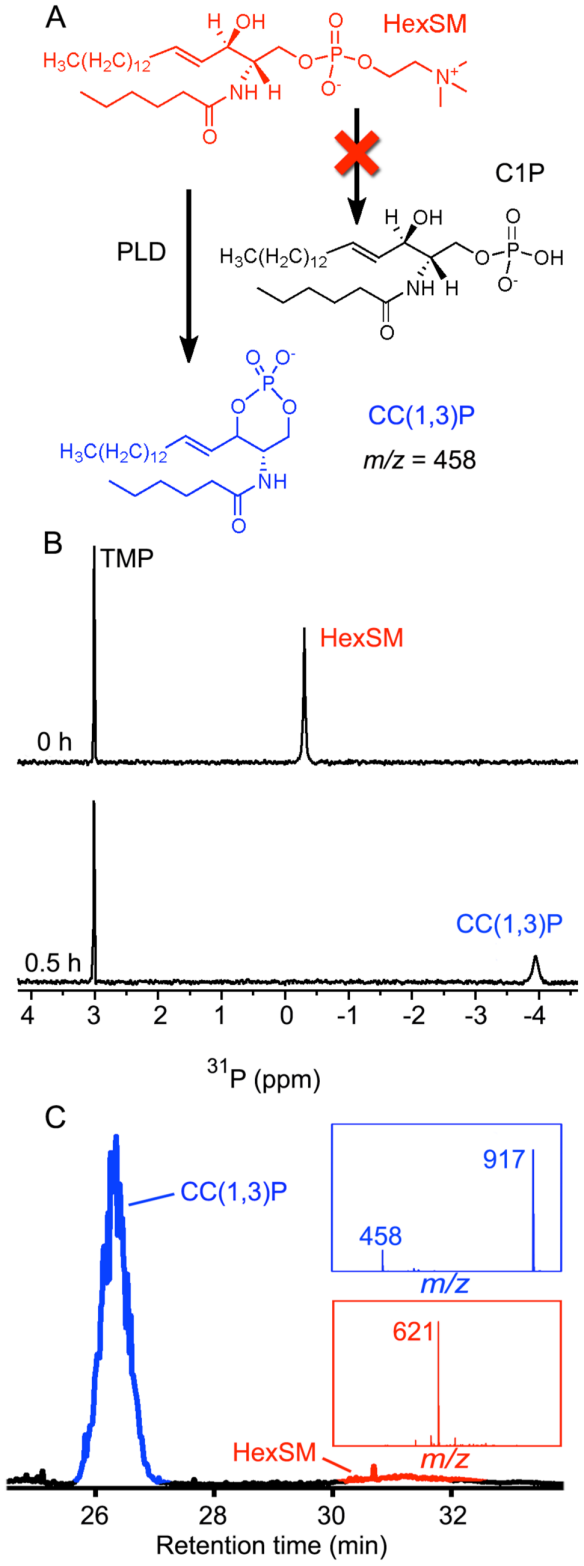
SicTox PLD enzymes from *Loxosceles* venoms convert sphingomyelin to cyclic ceramide phosphates. (A) Sphingomyelin (SM) is not converted not to ceramide-1-phosphate (C1P) but to a cyclic six membered ceramide(1,3)phosphate [CC(1,3)P]. (B) ^31^P-NMR spectra of 4 mM hexanoyl SM (HexSM) micelles, before (top) and 30 min after (bottom) addition of *L. arizonica* venom. The cyclic product persisted for over 12 h, and no monoester product (i.e. C1P) was detected. Analogous results were observed with purified SicTox αIB2bi enzyme. (C) LC-MS characterization of the product from the NMR sample in (B), showing results analogous to those shown in Figure 2B for conversion of LPC to CPA.

To determine the generality of the observed cyclization with LPC substrate, we also examined the reaction of recombinant αIB2bi and *L. arizonica* whole venom with sphingomyelin. Both αIB2bi and *L. arizonica* venom cleave choline from sphingomyelin [8], [17]. If the enzyme catalyzes cyclization of SM in a reaction analogous to the cyclization of LPC, we would expect the formation of a six-membered ring (Figure 4A). Indeed, results of ^31^P NMR assays with purified αIB2bi and natural sphingomyelin parallel those with palmitoyl LPC (Figure S3). We observe a small amount of unidentified product with a chemical shift that is inconsistent with ceramide-1-phosphate (C1P) (Figure S4B), the expected product of sphingomyelinase D (SMase D) activity via a hydrolysis reaction [24]. The product of turnover of αIB2bi with SM instead has a chemical shift near −4 ppm, consistent with the region expected for a six-membered ring cyclic phosphate [25]. As with the degradation of LPC, we observed precipitation.

We mitigated the product insolubility observed with natural SM by switching to a shorter acyl chain (hexanoyl) sphingomyelin (HexSM). Both recombinant αIB2bi and whole *L. arizonica* venom efficiently degrade HexSM to the cyclic ceramide(1,3)phosphate (CC(1,3)P) product (Figure 4B). The ^31^P resonance of the product is somewhat broad and the apparent yield as measured by signal integration is 42%, suggesting that shortening of the acyl chain does not entirely ameliorate product insolubility. Nevertheless, LC-MS analysis of the product (Figure 4C) reveals two peaks in the TIC-detected chromatograms: one at 26 min and a broader peak at ∼31 min. The peak at 26 min contains two species with *m/z* of 458 and 917, which, by analogy to CPA, we assign as [CC(1,3)P]^-^ and the corresponding [2CC(1,3)P+H^+^]^–^ dimer. The substrate *m/z* is consistent with [HexSM+acetate]^–^. These results show that production of cyclic phosphate and choline by the SicTox enzymes occurs for both sphingolipid and lysophospholipid substrates.

## Discussion

PLD activity with purified SicTox enzymes is often assayed by measuring the release of choline product from either LPC or SM [17]. Up to now, the identity of the second product has been assumed to be the monoester phosphate that would result from substrate hydrolysis [26]. LPA formation from SicTox enzymes has been reported using TLC detection [12], an enzyme-linked colorimetric assay [10], [27], and a cellular assay involving detection of 1-oleoyl LPA by human LPA_1_ receptor protein [14]. Notably, none of these methods involves direct, specific spectroscopic identification of LPA and none clearly rules out a cyclic phosphodiester product. For example, 1-oleoyl CPA activates human LPA_1_ receptor almost as well as 1-oleoyl LPA does [28], [29]. Similarly, the suggestion of C1P formation from SM is largely based on thin layer chromatography (TLC) [11]–[13] and a spectrophotometric method using a colored SM derivative [6], [18]. One previous ^31^P NMR study of SMase D activity by a purified SicTox enzyme reported only substrate decay, presumably because of product insolubility similar to that observed here [30]. A second ^31^P NMR study, which we came across after conducting our own experiments, actually did report ceramide-1-phosphate as a directly observed product, but the chemical shift is near −3 ppm rather than +3 ppm, and closely matches that of the cyclic product we observe (Figure 4) [31]. Given the consistency between this finding and our own, we suggest that in all of the previous assays, cyclic phosphates were understandably mistaken for the expected monoester products, which are chemically similar.

PLD enzymes can catalyze cleavage of the scissile diester bond by either a hydrolysis or a transphosphatidylation reaction [32], [33]. Conversion of substrate to cyclic phosphate-containing products by SicTox enzymes is a type of transphosphatidylation in which a free hydroxyl group within the substrate acts as an internal nucleophile. Formation of CPA from LPC has been reported with human enzymes [34], [35] and with a PLD from *S. chromofuscus* [19], both unrelated to the spider toxins. The ability of the SicTox enzymes to cyclize SM to the CC(1,3)P product is, to our knowledge, the first report of a SMase D that catalyzes such a reaction.

The exclusive formation of five- and six-membered cyclic phosphate esters in our experiments explains why all known phospholipid substrates of SicTox enzymes from *Loxosceles* species have a free hydroxyl at the C-2 or C-3 of the glycerol or sphingosine backbone [10]. Interestingly, the glycerophosphodiester phosphodiesterases (GDPD), which share an evolutionary history with the SicTox enzymes [24], [36], act on substrates that contain a free hydroxyl at both the C-2 or C-3 positions of the glycerol backbone. In fact, one proposed catalytic mechanism for the GDPD enzymes invokes formation of a cyclic intermediate [37]. Moreover, some GDPD enzymes can form a cyclic phosphodiester as a final product, rather than as an intermediate [38]. Therefore, our unanticipated results are completely consonant with the known substrate specificity and evolutionary history of these enzymes.

The etiology of loxoscelism is still poorly understood [2], but introduction of the SicTox toxins into mammalian tissue activates numerous cellular processes, for example the complement system and matrix metalloproteinase production [4]. Our results open up the prospect that the cyclic phosphates CPA and CC(1,3)P, produced by these toxins, directly contribute to the pathology of brown spider envenomation and may act as chemical triggers. For example, CPA is a known signaling molecule with distinct agonistic effects from LPA on mammalian cells [39], and is an inhibitor of peroxisome proliferator-activated receptor gamma, a nuclear receptor that helps to regulate cell proliferation, apoptosis, and inflammation [40]. Also, conversion of SM to CC(1,3)P may disturb bilayer integrity and morphology [41], which could affect membrane asymmetry. Loss of membrane asymmetry has been proposed as a mechanism to induce the complement pathway of the innate immune response, which can result in cell lysis [4], [6], [42]. Although cyclic phosphate esters could undergo ring opening in biological systems to yield monoester phosphates, CC(1,3)P is resistant to lysis when exposed to living cells [43] and CPA has been estimated to exist in human serum at a concentration of 0.1 µM based on isolation from albumin samples [22]. Thus, the products of the *Loxosceles* PLD toxins reported in our work may persist *in* *vivo*. Work with living cells and/or animal tissue may help determine if the putative accumulation of cyclic phospholipid products *in vivo* contributes to the necrotic state. Future research on the biological effects of cyclic phospholipids may shed light on the molecular basis of loxoscelism, as well as any possible function these products may play in their natural role of capture and immobilization of arthropod prey by brown spiders.

## Supporting Information

Figure S1 **Heterologous expression of αIB2bi proteins and assay to determine enzymatic activity.** **A:** Nonreducing SDS-PAGE analysis of purification of recombinant wild-type αIB2bi and an H47N variant by nickel affinity chromatography. The labels refer to cleared lysate (Load), flow through (FT), wash (W1 and W2), and eluate (E1 and E2) fractions. **B:** A colorimetric assay of the fractions from **A** using sphingomyelin as substrate. **C:** the same assay as **B** except with palmitoyl LPC as substrate. The assay detects choline release from substrate.
(PDF)

Figure S2 **^31^P-NMR spectra of commercially available palmitoyl lysophospholipids in borate buffer.** **A:** 1-Palmitoyl-2-hydroxy-*sn*-glycero-3-phosphocholine (16∶0 LPC, 4 mM), the diester phosphate substrate. **B:** 1-palmitoyl-2-hydroxy-*sn*-glycero-3-phosphate (16∶0 LPA, 4 mM), the putative monoester phosphate product from *Loxosceles* PLD toxins. **C:** mixture of 16∶0 LPC and 16∶0 LPA (2 mM each). **D:** mixture of 16∶0 LPC (3 mM) and 1-palmitoyl-*sn*-glycero-2,3-cyclic-phosphate (16∶0 CPA, 2 mM), a mixture of substrate and cyclic product (see also **Figure 1**). Trimethyl phosphate (TMP; 1 mM) was added to each sample as an internal chemical shift and concentration standard.
(PDF)

Figure S3 **Degradation of natural SM (4 mM; chicken egg) by αIB2bi enzyme monitored by 31P-NMR in borate buffer.** After 24 h, the only detectable product resonance (−3.7 ppm) is consistent with a cyclic phosphate containing a six-membered ring: cyclic ceramide(1,3)phosphate (CC(1,3)P). After 48 h, nearly all the SM substrate was consumed, but the product resonance remained weak. White precipitate was observed, suggestive of product insolubility. A chemical shift indicative of a monoester phosphate product of ceramide-1-phosphate was never observed (see **Figure S4**). SM micelles also included 12 mM Triton X-100 detergent. Trimethyl phosphate (TMP; 1 mM) was added as a chemical shift and concentration standard.
(PDF)

Figure S4 **^31^P-NMR spectra of commercially available sphingomyelin (SM) derived from chicken egg and N-oleoyl-ceramide-1-phosphate (18∶1 C1P) in borate buffer.** **A:** 4 mM SM with 12 mM Triton X-100. **B:** 0.5 mM SM, 0.5 mM 18∶1 C1P and 4 mM Triton X-100. Trimethyl phosphate (TMP; 1 mM) was added as a chemical shift and concentration standard.
(PDF)
