# Supplementary figures and images for: Phospholipase D Toxins of Brown Spider Venom Convert Lysophosphatidylcholine and Sphingomyelin to Cyclic Phosphates
Source: PLoS One. 2013 Aug 29;8(8):e72372. doi: 10.1371/journal.pone.0072372 (PMC3756997; doi:10.1371/journal.pone.0072372)

**A**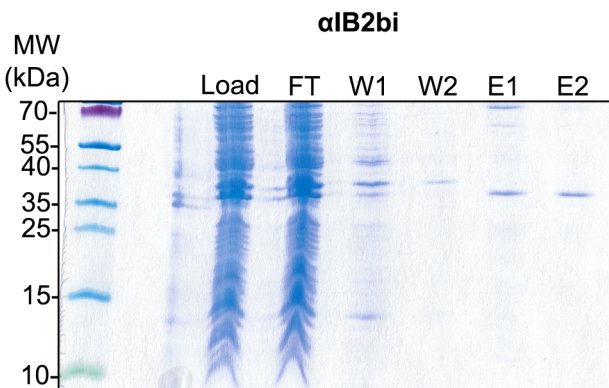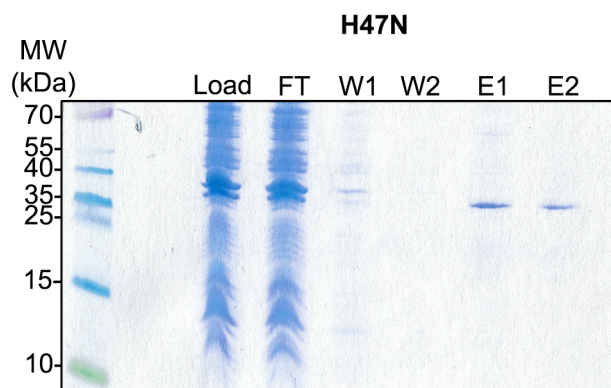**B**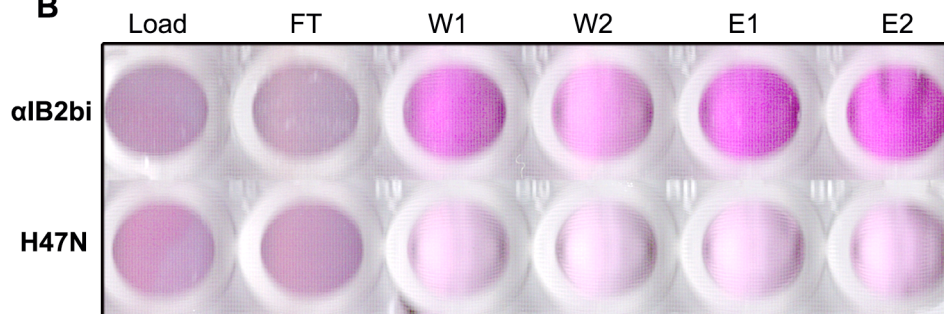**C**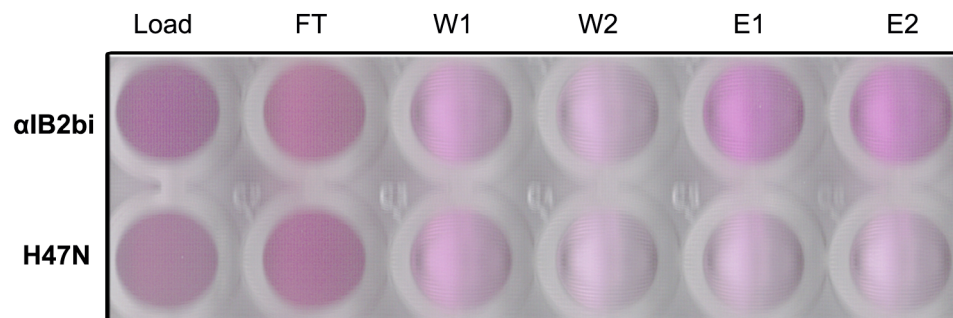

Supplement: Figure S1 — Heterologous expression of αIB2bi proteins and assay to determine enzymatic activity. A: Nonreducing SDS-PAGE analysis of purification of recombinant wild-type αIB2bi and an H47N variant by nickel affinity chromatography. The labels refer to cleared lysate (Load), flow through (FT), wash (W1 and W2), and eluate (E1 and E2) fractions. B: A colorimetric assay of the fractions from A using sphingomyelin as substrate. C: the same assay as B except with palmitoyl LPC as substrate. The assay detects choline release from substrate. (PDF) [file pone.0072372.s001.pdf]

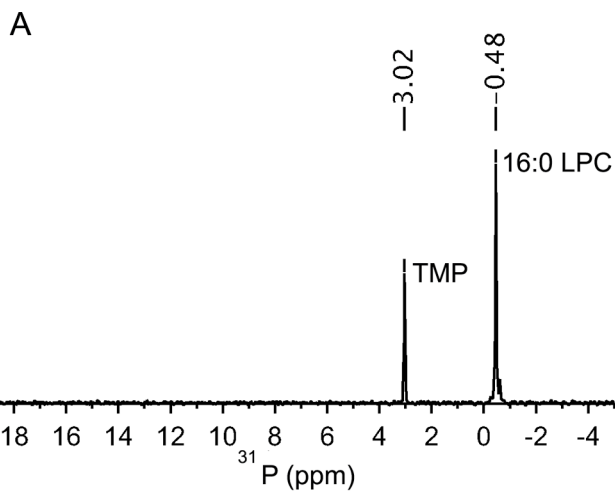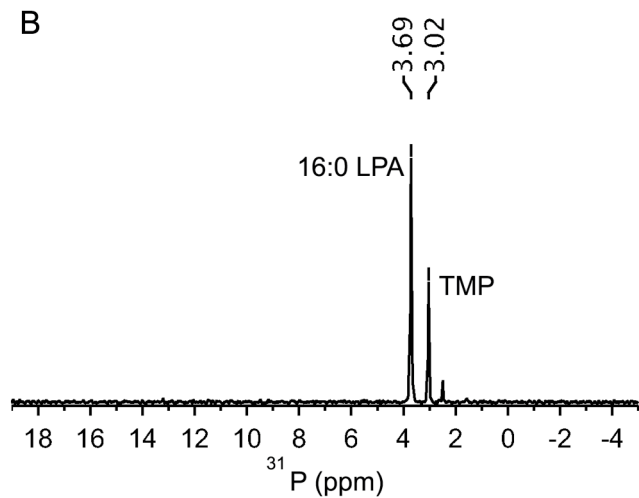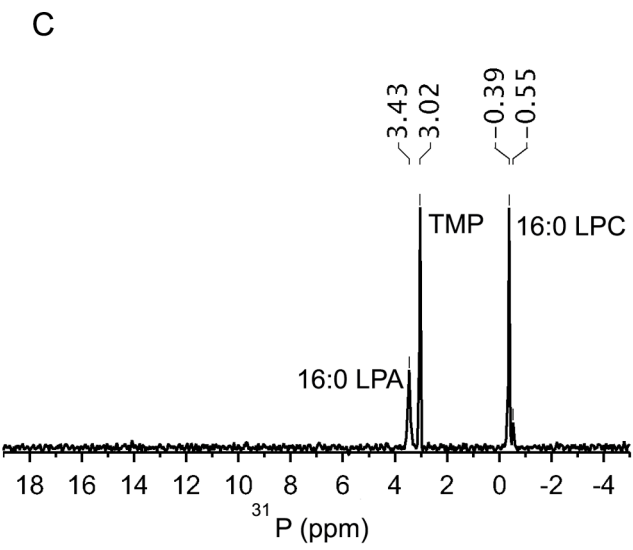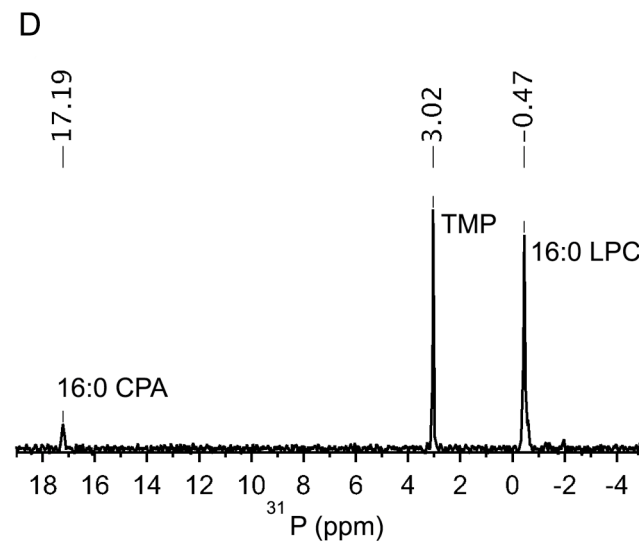

Supplement: Figure S2 — 31P-NMR spectra of commercially available palmitoyl lysophospholipids in borate buffer. A: 1-Palmitoyl-2-hydroxy-sn-glycero-3-phosphocholine (16∶0 LPC, 4 mM), the diester phosphate substrate. B: 1-palmitoyl-2-hydroxy-sn-glycero-3-phosphate (16∶0 LPA, 4 mM), the putative monoester phosphate product from Loxosceles PLD toxins. C: mixture of 16∶0 LPC and 16∶0 LPA (2 mM each). D: mixture of 16∶0 LPC (3 mM) and 1-palmitoyl-sn-glycero-2,3-cyclic-phosphate (16∶0 CPA, 2 mM), a mixture of substrate and cyclic product (see also Figure 1 ). Trimethyl phosphate (TMP; 1 mM) was added to each sample as an internal chemical shift and concentration standard. (PDF) [file pone.0072372.s002.pdf]

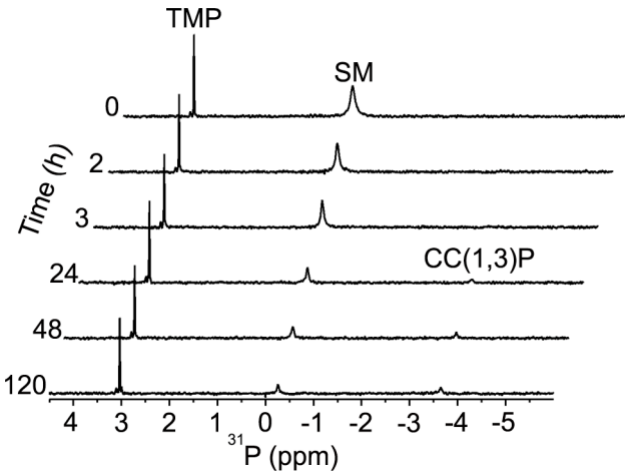

Supplement: Figure S3 — Degradation of natural SM (4 mM; chicken egg) by αIB2bi enzyme monitored by 31P-NMR in borate buffer. After 24 h, the only detectable product resonance (−3.7 ppm) is consistent with a cyclic phosphate containing a six-membered ring: cyclic ceramide(1,3)phosphate (CC(1,3)P). After 48 h, nearly all the SM substrate was consumed, but the product resonance remained weak. White precipitate was observed, suggestive of product insolubility. A chemical shift indicative of a monoester phosphate product of ceramide-1-phosphate was never observed (see Figure S4). SM micelles also included 12 mM Triton X-100 detergent. Trimethyl phosphate (TMP; 1 mM) was added as a chemical shift and concentration standard. (PDF) [file pone.0072372.s003.pdf]

A

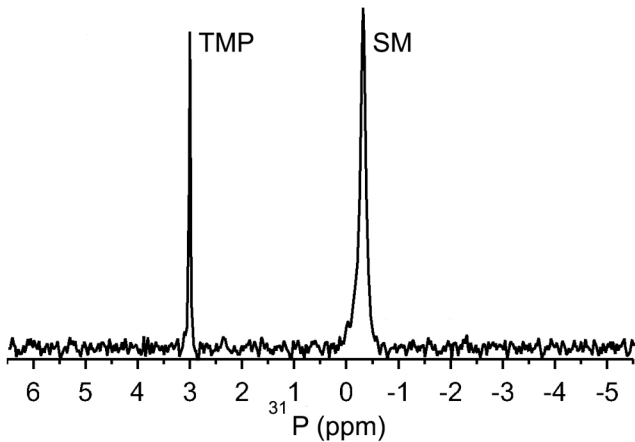

B

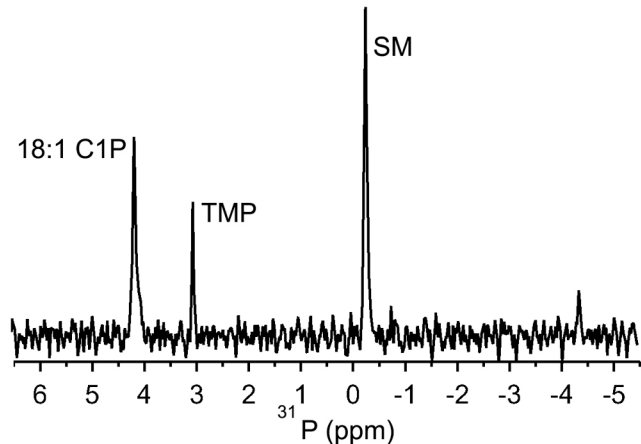

Supplement: Figure S4 — 31P-NMR spectra of commercially available sphingomyelin (SM) derived from chicken egg and N-oleoyl-ceramide-1-phosphate (18∶1 C1P) in borate buffer. A: 4 mM SM with 12 mM Triton X-100. B: 0.5 mM SM, 0.5 mM 18∶1 C1P and 4 mM Triton X-100. Trimethyl phosphate (TMP; 1 mM) was added as a chemical shift and concentration standard. (PDF) [file pone.0072372.s004.pdf]
